# A systematic review and meta-analysis of the association between age and degrees of avoidant decision-making style

**DOI:** 10.1007/s10433-025-00887-5

**Published:** 2025-10-15

**Authors:** Tarren Leon, Gabrielle Weidemann, Phoebe E. Bailey

**Affiliations:** 1 Graduate School of Health, University of Technology Sydney, Ultimo Broadway, Sydney, NSW 2007 Australia; 2 School of Psychology, Western Sydney University, Bankstown, Australia; 3 MARCS Institute for Brain, Behaviour, and Development, Western Sydney University, Westmead, Australia

**Keywords:** Avoidance, Avoidant decision-making, Decision-making style, General decision-making styles, Melbourne decision-making questionnaire

## Abstract

Initial empirical evidence and theories suggest that decision-making may become more avoidant with age. However, recent studies provide inconsistent evidence for this effect. We conducted a systematic review and meta-analysis of 19 studies (*N *= 7969) to assess evidence for an association between age and avoidant decision-making style. We included studies that used the avoidant subscale of the General-Decision-Making Style (GDMS) questionnaire or the buck-passing subscale of the Melbourne Decision-Making Questionnaire (complete avoidance), or the dependent subscale of the GDMS (partial avoidance). We also assessed potential moderators of the effect, including age range for each sample, gender, culture, participant sample type, publication year, decision style subscale, and degree of avoidance (complete vs. partial). Surprisingly, the data revealed a small association between older age and less avoidant decision-making style. Moderator analysis revealed that this association applied to complete decision avoidance (the avoidant and buck-passing subscales) and not partial avoidance (the dependent subscale). Additionally, moderation by sample type suggests that decision style does not become less avoidant until after middle age. We discuss important future directions for research aimed at investigating nuances that may contribute toward avoidant decision-making style in older age.

**Supplementary Information:**

The online version contains supplementary material available at 10.1007/s10433-025-00887-5.

## Introduction

Decision style is an individual attribute that refers to the predominant patterns a person displays in response to a decision, and can influence decision outcomes (Scott and Bruce 1995). The decision styles proposed by Scott and Bruce (1995) include avoidant, dependent, rational, spontaneous, and intuitive styles. These styles are not mutually exclusive, and can vary depending on the decision context, and individual differences such as cognitive abilities and habits (Thunholm 2004). However, as we age, we experience changes in cognitive abilities and emotion regulation that influence decision-making (Bruine de Bruin et al. 2012). Previous research indicates that older adulthood may be associated with avoidant decision-making to a greater extent than earlier stages of adulthood (Mather 2006), and the evidence base, in terms of avoidance as a decision-making style, has grown in recent years. A synthesis of existing data would provide a more comprehensive picture of age-related changes.

The avoidant decision style subscale in Scott and Bruce’s (1995) General-Decision-Making Style (GDMS) questionnaire assesses the general tendency to avoid or put off decisions. Their dependent decision style subscale assesses the tendency to search for advice, or direction/support from others when making decisions. The Melbourne Decision-Making Questionnaire (MDMQ) is a measure of decision approach—that is, the way an individual manages processes when experiencing decisional conflict (Mann et al. 1997). The questionnaire not only measures the ways individuals generally approach decisions but also reflects their styles of thinking. According to Mann et al. (1997), the buck-passing subscale of the MDMQ is a measure of defensive avoidance. Defensive avoidance includes a variety of strategies to avoid decisions, including behavioral (e.g., procrastinating, and passing decisions on to others to make) and mental (e.g., avoiding the realities of the decision) strategies. The MDMQ buck-passing subscale assesses complete decision avoidance.

A dual-process theory of age and decision-making (Peters et al. 2007) predicts preservation of the automatic, affective/experiential profile with increase in age relative to declines in the more effortful and deliberative, independent/self-controlled profile (however, see Keren (2013) for criticisms of dual-process theories). The theory is based on evidence that cognitive abilities such as executive functioning and working memory decline with age, while more automatic emotional processes remain intact or improve (MacPherson et al. 2002; Mikels and Taullahu, 2023). Currently, there exists limited research that specifically investigates the association between age and decision style, and findings have been mixed. Fatima et al. (2020) showed that, among young and middle-aged adults, there was a positive association between age and both the avoidant and dependent decision-making styles. However, counter to the dual-process theory, Delaney et al. (2015) found that older adults were more likely to fit an independent/self-controlled decision-making style (characterized by low scores on all five decision styles with the dependent and spontaneous styles being the lowest), rather than a dependent profile (characterized by high scores on the dependent style, and lower scores on all other styles) or an affective/experiential profile (indicated by high scores on the spontaneous and intuitive styles).

Decision-making falls on a continuum from complete autonomy, to shared decision-making, to delegating decisions to another person, to completely avoiding the decision (Löckenhoff 2018). Compared to young adults, there is evidence that older adults are more likely to prefer to delegate decision-making to others (Finucane et al. 2002), or to engage in choice deferral (Chen et al. 2011). There is also some evidence to suggest that, relative to young adults, older adults rely more on advice in their decision-making (Bailey et al. 2021, although see Leon et al. 2024). This is notable because advice-taking reflects, in part, an avoidance strategy by sharing the responsibility of decision-making with the advisor (Harvey and Fischer 1997). Increased advice-taking provides some evidence for a more dependent and/or avoidant decision-making style in older age.

While the avoidant style subscale of the GDMS assesses complete decision avoidance, the GDMS dependent subscale indexes partial avoidance, and there is evidence that the two are positively correlated (among the studies included in the present meta-analysis, 11 studies reported correlations between 0.23 to 0.44, and two studies reported lower correlations of 0.05 and 0.08). The avoidant style subscale includes questions such as, “I postpone decision-making whenever possible” and “I put off making many decisions because thinking about them makes me uneasy.” These questions reflect choice deferral, which has been attributed to avoidance of possible negative emotions associated with a decision (Chen et al. 2011; Hallenbeck et al. 2022; Lauderdale et al. 2019). Items in the dependent subscale of the GDMS (e.g., “If I have the support of others, it is easier for me to make decisions”) may reflect age-related declines in some cognitive abilities, which result in compensatory strategies such as involving others in decision-making (Baltes and Baltes, 1990). The buck-passing subscale of the MDMQ includes questions measuring decision deferral/avoidance (e.g., “I avoid making decisions”) and preference to avoid decision responsibility (e.g., “I do not like to take responsibility for making decisions”). The current meta-analysis extends past research with a broader conceptualization of avoidant decision-making style which encompasses both complete avoidance and partial avoidance. We also assessed for possible moderation of the overall meta-analytic correlation between age and avoidant decision style by degree of avoidance (i.e., complete avoidance (the avoidant and buck-passing subscales) vs. partial avoidance (the dependent subscale)).

In line with Lӧckenhoff’s (2018) conceptual framework for mapping age differences in decision-making, it has been recommended that decision-making research examine contextual factors such as cohort or cultural differences (Appelt et al. 2011). Indeed, cultural background can shape the values, beliefs, and attitudes that guide decision-making (Dabić et al. 2014). It is possible that negative age stereotypes reduce older adults’ cognitive resources and/or their confidence in their cognitive resources, thus increasing decision avoidance (Lӧckenhoff, 2018). These negative stereotypes have become greater among Eastern (i.e., collectivist) relative to Western (i.e., individualist) cultures (Alonso Debreczeni and Bailey 2021). Hofstede (2001) refers to culture as collective, learned “programming of the mind,” acquired from an individual’s social environment. Hofstede Insights (2024) provides six dimensions (i.e., power distance, individualism vs collectivism, motivation toward achievement and success, uncertainty avoidance, long-term vs short-term orientation, and indulgence vs restraint) from which national cultures can be compared against each other. While previous research has investigated decision-making differences according to individualistic versus collectivistic cultures (LeFebvre and Franke 2013; Mann et al. 1998), to our knowledge, the research has not yet extended investigations to the remaining five dimensions, particularly in terms of how they may contribute to age-related differences in decision-making styles. As noted Lӧckenhoff’s (2018), framework also refers to potential effects of cohort as opposed to age, and as such, publication date was included as a potential moderator and a proxy measure for societal changes that may co-occur with aging.

The aim of the current systematic review and meta-analysis is to gain a better understanding of the association between age and avoidant decision-making styles. Additionally, the meta-analysis investigated potential moderators of this association, including age range to control for differing ranges of age in participant samples, the culture of the sample (i.e., the 6 Hofstede culture dimensions), the decision style subscale (i.e., dependence, avoidance, buck-passing), and publication date. As an exploratory analysis, we included participant sample type (i.e., university students, professionals, and community samples), and degree of avoidance (partial vs. complete) as possible moderators.

## Method

This meta-analysis and systematic review was conducted as per the Preferred Reporting Items for Systematic reviews and Meta-Analyses (PRISMA) statement (Page et al. 2021). Article screening was conducted using the web-based software Covidence (Veritas Health Innovation 2024). This study was preregistered at AsPredicted https://aspredicted.org/3nd5-t95x.pdf, and data are available at https://osf.io/6vrmh/.

### Literature search

**Fig. 1:**
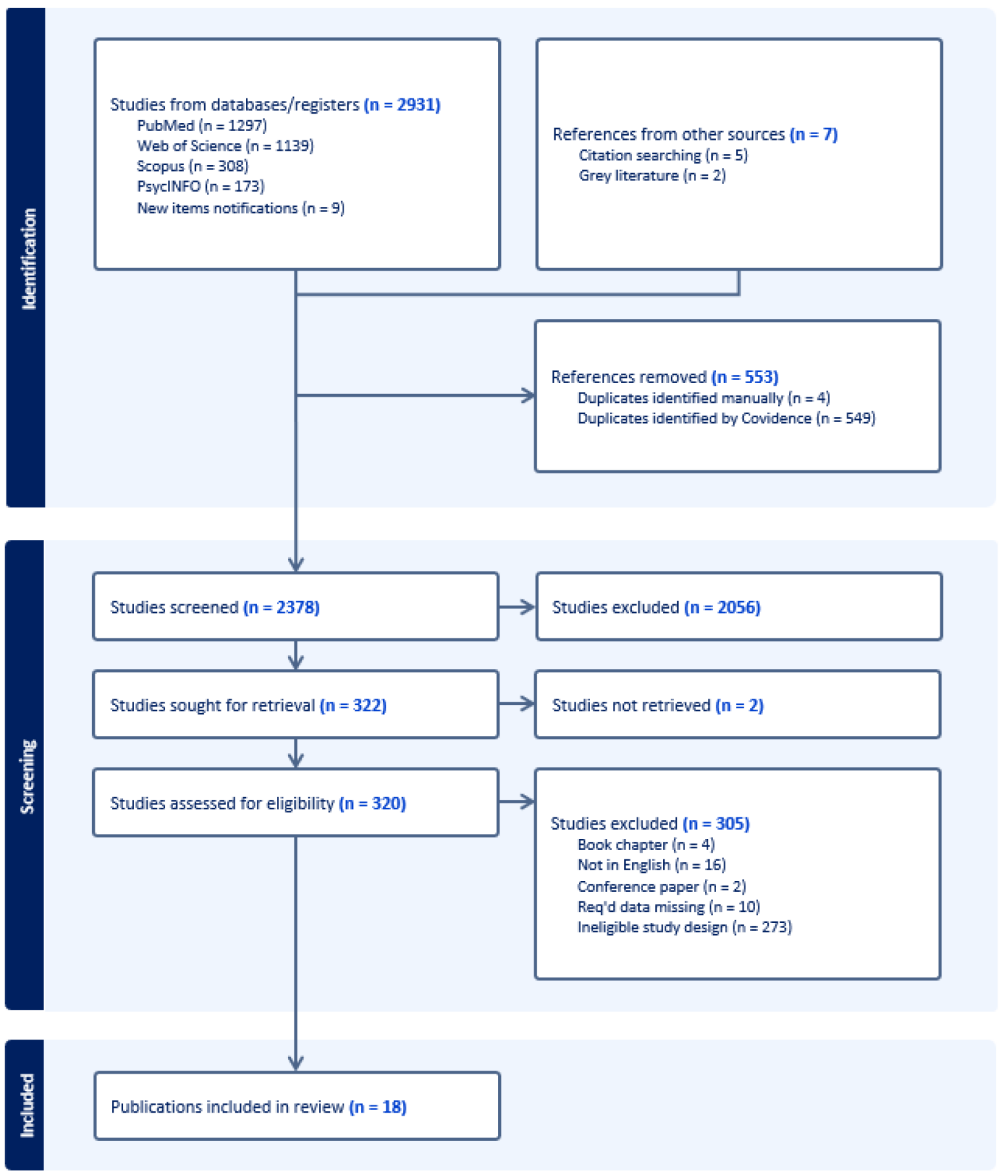
PRISMA diagram of data screening and selection process

A literature search was conducted on 5 July 2023, and new articles were considered up until data analysis on July 19, 2024. The databases PubMed, Web of Science, Scopus, and PsycINFO were used. Search terms were (decision-making OR decision) AND (style OR styles) AND (general decision-making styles OR GDMS OR Melbourne decision-making questionnaire OR MDMQ). The searches returned 1297, 1139, 308, and 173 possible studies, respectively, for inclusion. Manual forwards and backwards searches were also conducted on the final set of included studies. A PRISMA flowchart displays the process of exclusion and inclusion (Fig. 1).

### Eligibility criteria

To ensure accurate capture of studies, the criteria for the title and abstract screening included a mention of “decision-making style,” and studies in English. At the full-text screening, eligibility criteria were as per our preregistration plan and included: a measure of avoidant decision-making style (as per the GDMS or MDMQ), and precise statistics convertible to effect sizes. Initially, the eligibility criteria also included that the studies had a sample of young and older adults, where the older group had a mean age of at least 60 years, and the younger group had a mean age of at least 30 years younger than the mean age of the older group. It was expected that studies involving age differences would likely involve these extreme age group samples. Upon screening the studies, it was found that it was more common for studies to include a continuous variable of age. As such, the eligibility was extended so that studies with any adult age range would be included, as long as the precise age range, age mean, and age standard deviation of participants were reported.

### Screening

The first author extracted all data. The first author and a research assistant conducted the title and abstract screening, and full-text screening. Conflicts were resolved by the first author after discussion with the research team. Where the required data were not available in a paper, the corresponding or first author was contacted via email. Two attempts to contact authors were made, and studies were excluded when there was no response, or when the author advised that the data were no longer available or that the required data were not collected (e.g., age information was collected from participants via a closed question whereby participants had to select which age range they fell into versus an open question format in which participants provide their current exact age) (i.e., Akyürek 2020; Baiocco et al. 2009; Berisha et al. 2022; Bouckenooghe et al. 2007; Brown and Daus 2016; Dilawar et al. 2019; Hayee and Hassan 2011; Ozturk and Arikan 2020; Ugurlu, 2013; Umeh and Omari-Asor 2011; Verma et al. 2016). Following removal of duplicate studies (*N* = 553), 2056 studies were excluded at abstract and title screening, and 322 studies were assessed for eligibility based on a full-text review. This process is depicted in a PRISMA diagram in Fig. 1. The final extracted sample included 18 publications and 19 studies.

### Data extraction and effect size calculation

Table 1 presents the sample and task characteristics of each dataset. Avoidant decision-making was assessed using the avoidant and dependent subscales of the GDMS, and the buck-passing subscale of the MDMQ. The first author extracted data for effect sizes (avoidant decision-making *M* and *SD*s in the categorical age groups, or correlation coefficients between age and avoidant decision-making for continuous age). A second blind extraction was conducted to ensure reliability of the data.

**Table 1:** Sample and task characteristics for each dataset included in the meta-analysis

Study	Sample characteristics				Moderators								
	*N*	Mean (*SD*) Age	% Female	Country	Age rage	Culture: Power distance	Culture: Individualism	Culture: MTAS	Culture: UA	Culture: LTO	Culture: Indulgence	Participant sample type	Decision style subscale
AlAmer (2023) Group 1	103	33.95 (6.3) (Full sample)	97.6 (Full sample)	Saudi Arabia	25–29	72	48	43	64	27	14	Professional	Avoidance (GDMS)
AlAmer (2023) Group 1	103	33.95 (6.3) (Full sample)	97.6 (Full sample)	Saudi Arabia	25–29	72	48	43	64	27	14	Professional	Dependence (GDMS)
AlAmer (2023) Group 2	115	33.95 (6.3) (Full sample)	97.6 (Full sample)	Saudi Arabia	30–40	72	48	43	64	27	14	Professional	Avoidance (GDMS)
AlAmer (2023) Group 2	115	33.95 (6.3) (Full sample)	97.6 (Full sample)	Saudi Arabia	30–40	72	48	43	64	27	14	Professional	Avoidance (GDMS)
AlAmer (2023) Group 3	27	33.95 (6.3) (Full sample)	97.6 (Full sample)	Saudi Arabia	41–59	72	48	43	64	27	14	Professional	Avoidance (GDMS)
AlAmer (2023) Group 3	27	33.95 (6.3) (Full sample)	97.6 (Full sample)	Saudi Arabia	41–59	72	48	43	64	27	14	Professional	Avoidance (GDMS)
Aluja et al. (2023) Group 1	538	45.67 (16.07)	0	Spain	18–90	57	67	42	86	47	44	Community	Avoidance (GDMS)
Aluja et al. (2023) Group 1	538	45.67 (16.07)	0	Spain	18–90	57	67	42	86	47	44	Community	Dependence (GDMS)
Aluja et al. (2023) Group 2	453	44.04 (15.25)	100	Spain	18–90	57	67	42	86	47	44	Community	Avoidance (GDMS)
Aluja et al. (2023) Group 2	453	44.04 (15.25)	100	Spain	18–90	57	67	42	86	47	44	Community	Dependence (GDMS)
Berisha et al. (2018)	152	20.92 (0.95)	57.89	Kosovo	20–23	NA	NA	NA	NA	NA	NA	University students	Avoidance (GDMS)
Berisha et al. (2018)	152	20.92 (0.95)	57.89	Kosovo	20–23	NA	NA	NA	NA	NA	NA	University students	Dependence (GDMS)
Caldera (2018)	312	42.85 (12.80)	67.00	US	18–79	40	60	62	46	50	68	Community	Avoidance (GDMS)
Caldera (2018)	312	42.85 (12.80)	67.00	US	18–79	40	60	62	46	50	68	Community	Dependence (GDMS)
Calleja et al. (2020)	52	31.79 (4.7)	0	Australia	24.5–47.5	38	73	61	51	56	71	Professional	Avoidance (GDMS)
Calleja et al. (2020)	52	31.79 (4.7)	0	Australia	24.5–47.5	38	73	61	51	56	71	Professional	Dependence (GDMS)
Curşeu & Schruijer (2012)	102	41.72 (6.05)	25.49	Netherlands	28–54	38	100	14	53	67	68	Professional	Avoidance (GDMS)
Curşeu & Schruijer (2012)	102	41.72 (6.05)	25.49	Netherlands	28–54	38	100	14	53	67	68	Professional	Dependence (GDMS)
Da Lama & Brenlla (2023)	209	32.84 (8.73)	77.03	Argentina	19–64	49	51	56	86	29	62	Community	Buck-passing (MDMQ)
Delaney et al. (2015)	1066	53.49 (14.85)	56.20	US	18–93	40	60	62	46	50	68	Community	Avoidance (GDMS)
Delaney et al. (2015)	1066	53.49 (14.85)	56.20	US	18–93	40	60	62	46	50	68	Community	Dependence (GDMS)
Erceg & Galić (2024) Study 2	210	34.31 (10.63)	49.05	Croatia	19–63	73	42	40	80	40	33	Professional	Avoidance (GDMS)
Erceg & Galić (2024) Study 2	210	34.31 (10.63)	49.05	Croatia	19–63	73	42	40	80	40	33	Professional	Dependence (GDMS)
Erceg & Galić (2024) Study 3	53	47.15 (10.66)	35.85	Croatia	27–69	73	42	40	80	40	33	Professional	Avoidance (GDMS)
Erceg & Galić (2024) Study 3	53	47.15 (10.66)	35.85	Croatia	27–69	73	42	40	80	40	33	Professional	Dependence (GDMS)
Fatima et al. (2020)	195	43.82 (8.67)	26.15	Pakistan	30–59	55	5	50	70	19	0	Professional	Avoidance (GDMS)
Fatima et al. (2020)	195	43.82 (8.67)	26.15	Pakistan	30–59	55	5	50	70	19	0	Professional	Dependence (GDMS)
Kornilova et al. (2018) Group 1	79	18.48 (1.33)	63.29	Azerbaijan	16–22	85	28	50	88	59	22	University students	Buck-passing (MDMQ)
Kornilova et al. (2018) Group 2	195	19.62 (1.14)	81.03	Russia	18–28	93	46	36	95	58	20	University students	Buck-passing (MDMQ)
Leon, Weidemann & Bailey (2025a)	132	49.65 (18.58)	50.76	Australia	19–89	38	73	61	51	56	71	Community	Avoidance (GDMS)
Leon, Weidemann & Bailey (2025a)	132	49.65 (18.58)	50.76	Australia	19–89	38	73	61	51	56	71	Community	Dependence (GDMS)
Leon, Weidemann, Kneebone & Bailey (2025b)	179	52.01 (16.77)	45.25	Australia	21–89	NA	NA	NA	NA	NA	NA	Community	Avoidance (GDMS)
Leon, Weidemann, Kneebone & Bailey (2025b)	179	52.01 (16.77)	45.25	Australia	21–89	NA	NA	NA	NA	NA	NA	Community	Dependence (GDMS)
Loo (2000)	223	23.67 (5.21)	41.70	Canada	19–50	39	72	52	48	54	68	University students	Avoidance (GDMS)
Loo (2000)	223	23.67 (5.21)	41.70	Canada	19–50	39	72	52	48	54	68	University students	Dependence (GDMS)
Parker et al. (2007)	360	47.70 (17.00)	73.80	US	18–88	40	60	62	46	50	68	Community	Avoidance (GDMS)
Parker et al. (2007)	360	47.70 (17.00)	73.80	US	18–88	40	60	62	46	50	68	Community	Dependence (GDMS)
Phillips & Reddie (2007)	90	36.70 (19.30)	71.00	Australia	18–75	38	73	61	51	56	71	Professional	Buck-passing (MDMQ)
Urieta et al. (2021)	1562	40.03 (18.43)	54.30	Spain	18–88	57	67	42	86	47	44	University and community	Buck-passing (MDMQ)
Urieta et al. (2023)	1562	40.02 (18.43)	54.30	Spain	18–90	57	67	42	86	47	44	University students and community	Avoidance (GDMS)
Urieta et al. (2023)	1562	40.02 (18.43)	54.30	Spain	18–90	57	67	42	86	47	44	University students and community	Dependence (GDMS)

MTAS = *MTAS* Motivation toward achievement and success, *UA* Uncertainty avoidance, *LTO* Long-term orientation

For extractions with age groups, each age group was included. Following Bagaïni et al.’s (2023) processes for calculating effect sizes for extreme age group and continuous age designs, the standardized mean difference between each age group was converted into a point-biserial correlation coefficient. If a Pearson’s *r* correlation coefficient between age groups and avoidant decision-making was available, this was used instead. For studies where age and the avoidant decision-making outcomes were continuous, Pearson’s *r* correlation coefficient was used. From the 19 studies (of the 18 publications), 41 effect sizes were extracted.

Effect sizes were coded so that increasing scores on avoidant decision-making were indicated by greater effect size values. As per Bailey and Leon (2019), at least five effect sizes were needed in each categorical level for a moderator to be included in moderator analyses. The following moderators were thus investigated for their relationships with the effect sizes: a) age range—calculated so that for Pearson’s *r* correlations, the age difference in years between the youngest and oldest participants was used, and for point-biserial correlation the mean age of the youngest and oldest groups was used; b) gender (% female); c) culture of the sample, as indicated by Hofstede’s dimensions of power distance (i.e., a culture’s attitude toward inequalities within the society), individualism (i.e., the extent people of a society have a self-image defined in terms of “I” or “We”), motivation toward achievement and success (i.e., how driven the society is by competition, achievement, and success (higher scores), or by caring for others and quality of life (lower scores)), uncertainty avoidance (i.e., the extent societies are controlling or accepting of an unknown future), long-term orientation (i.e., how suspicious or encouraging societies are toward societal change), and indulgence (i.e., the extent of control people have over their desires and impulses); d) the measure of avoidant decision-making (i.e., avoidant or dependent subscale of the GDMS, or buck-passing subscale of the MDMQ); e) participant sample type (i.e., students, professionals, and community); f) effect size metric (i.e., point-biserial correlation, or Pearson’s *r* correlation coefficient), and g) degree of avoidant decision-making style (i.e., complete or partial avoidance). In instances of a combination of participant sample types (i.e., Urieta et al. 2021 and Urieta et al. 2023), the study was not included. Given that the topic of decision (as in our preregistration) is not specified in the avoidant, dependent, and buck-passing subscales, this was unable to be included. Continuous moderator variables were grand mean centered.

### Meta-analytic approach

Analyses were conducted in R (R Core Team 2021), using the metafor (Viechtbauer 2010), clubSandwich (Pustejovsky 2024), and TOSTER (Caldwell 2022; Lakens 2017) packages. Analysis code was modeled after that used by Bagaïni et al. (2023), and is available at https://osf.io/6vrmh/.

A three-level meta-analysis model was fitted, with restricted maximum likelihood estimation as outlined in Assink and Webbelink (2016). Random effects were included at the individual (level 1), within studies (level 2), and between studies (level 3) levels, as per Van den Noortgate et al. (2013). This allowed for correlation between sampling errors of the studies and provided a method to address dependence of effect sizes (i.e., the violation of the assumption that effect sizes are independent), which can occur when individual studies have multiple effect sizes. A correlation of 0.5 was used (where 0 would indicate outcomes are independent, and 1 would indicate complete correlation), and sensitivity analyses were performed on correlations varying between 0.1 and 0.9. Robust variance estimation methods were also performed to manage possible unknown forms of dependency (Pustejovsky and Tipton 2022).

The meta-analytic effect size estimate was tested for statistical significance with alpha set to 0.05, and two one-sided equivalence tests were also conducted to determine how *meaningful* the result was—that is, the effect size was tested to determine whether it fit within the highest and lowest bounds of the smallest effect size of interest (Lakens et al. 2018). The observed effect can be rejected if the upper or lower z-scores (i.e., [effect size + higher bound of smallest effect size of interest] / standard error; [effect size—lower bound of smallest effect size of interest] / standard error) fall within the bounds of the smallest effect size of interest (Lakens et al. 2018; Rogers et al. 1993). Meta-analyses have sufficient statistical power for narrow equivalence bounds of *r* = − 0.1 and *r* = 0.1 (Lakens 2017), thus *r* = [0.1] was selected as the smallest effect size of interest. According to recommendations by Brydges (2019), Pearson’s *r* = 0.10, *r* = 0.20, and *r* = 0.30 are considered small, to moderate, and large effects, respectively.

After investigating the overall effect size, separate meta-regression models were run to examine the potential moderators of the association between avoidant decision-making and age.

## Results

### Overall effect

**Fig. 2:**
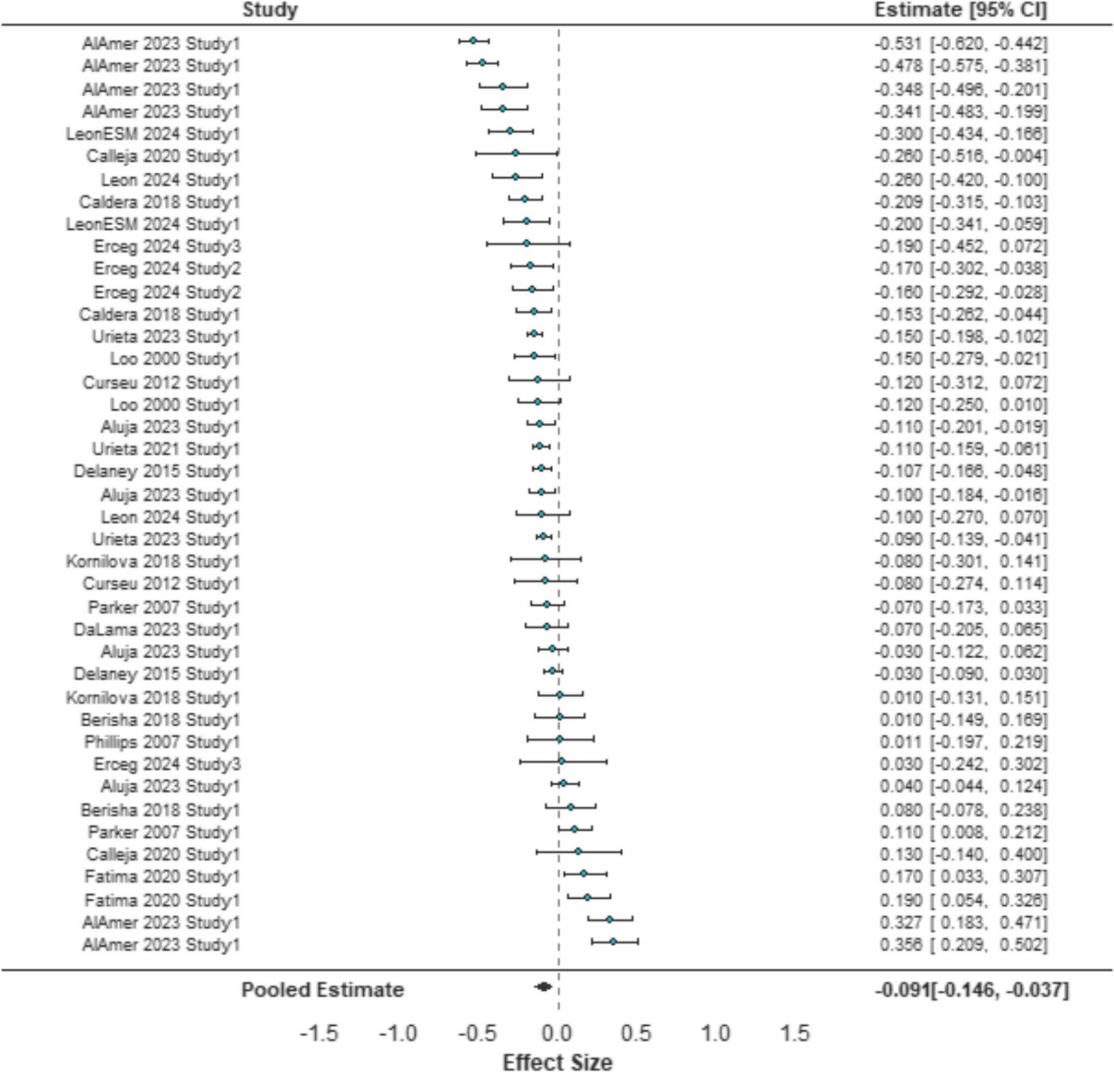
A forest plot of age and avoidant decision-making style. *Note*: the diamond represents the summary pooled effect size of age and avoidant decision-making style with 95% confidence intervals ordered by effect size

Our three-level meta-analysis of all effect sizes (*k* = 41) showed that age is negatively associated with avoidant decision-making (*r* = − 0.09, *SE* = 0.03, 95% CI [− 0.15, − 0.04], *p* = 0.003, as is presented in Fig. 2. A boxplot indicated four outlier effect sizes (− 0.478, − 0.531, 0.356, 0.327). The removal of the data points did not significantly change the overall effect (*k* = 36, *r* = − 0.09, *SE* = 0.02, 95% CI [− 0.13, − 0.05], *p* < 0.0001), and so they were retained in the subsequent analyses. The equivalence bounds test indicated that the effect was not within the bounds of the smallest effect size of interest, *r* = [0.1], (90% CI [− 0.13, − 0.05], *p* = 0.365).

As per our preregistration, the fit of the three-level model was compared against two two-level models, one in which the within-study variance (level 2) was not modeled, and another in which the variance between studies (level 3) was excluded. Likelihood-ratio tests showed that the three-level model was a better fit than either of the two two-level models (*p*s < 0.0001), indicating significant heterogeneity between the studies. The estimated variance components for between and within studies were *τ*^2^ = 0.033 and *τ*^2^ = < 0.001, respectively. Of the total variance, 8% was attributed to variance at level 1 (sampling variance), < 0.001% at level 2 (within-studies variance), and 92% attributed to variance at level 3 (between studies). The potential influence of moderators (i.e., age range, gender, culture dimensions, measure of decision-making style, participant sample type, effect size metric, and publication year) was thus investigated in separate three-level meta-regressions.

The overall effect was moderated by type of effect size metric, such that effect size calculated from Pearson’s *r* correlation coefficients is negative (*k* = 35, *r* = − 0.08, 95% CI [− 0.13, − 0.03], *p* = 0.007), while there is no significant effect based on point-biserial correlations (*k* = 6, *r* = − 0.19, 95% CI [− 0.61, 0.22], *p* = 0.106). Effect size metric may have driven the age effect; however, given that 85% of the effect sizes were calculated using Pearson’s *r* correlation coefficients, we treat this difference with caution.

We found a significant moderator of participant sample type, such that there is a negative association between age and avoidant decision-making within community samples (*k* = 15, *r* = − 0.10, 95% CI [− 0.19, − 0.01], *p* = 0.033), but not professional samples (*k* = 17, *r* = − 0.09, 95% CI [− 0.25, 0.07], *p* = 0.203) or university student samples (*k* = 6, *r* = − 0.04, 95% CI [− 0.29, 0.19], *p* = 0.513). As only 15% of the effect sizes included university student samples, this difference is also treated with caution. Pairwise comparisons between university students and community participants, between university students and professional participants, and between community and professional participants did not result in any significant differences (*p* = 0.357, *p* = 0.617, and *p* = 0.811, respectively).

The overall effect was also found to be moderated by the degree of avoidance. A negative effect was found for complete avoidance (*k* = 23, *r* = − 0.12, 95% CI [− 0.22, − 0.02], *p* = 0.022), but not for partial avoidance (*k* = 19, *r* = − 0.06, 95% CI [− 0.13, 0.02], *p* = 0.111). This suggests that the age effect may be specific to a completely avoidant decision-making style.

The results of nonsignificant moderators are reported in the Supplementary Materials.

### Publication bias and power

Publication bias was tested using one pooled estimate of age and avoidant decision style for each study. Among studies with dependent outcomes, effect sizes for each outcome were pooled. The MAd package (Del Re and Hoyt 2014) was used in R to calculate the composite estimate, using a conservative correlation of 1.0 among within-study outcomes. Borenstein et al. (2009) procedures were followed for aggregating dependent effect sizes.

**Fig. 3:**
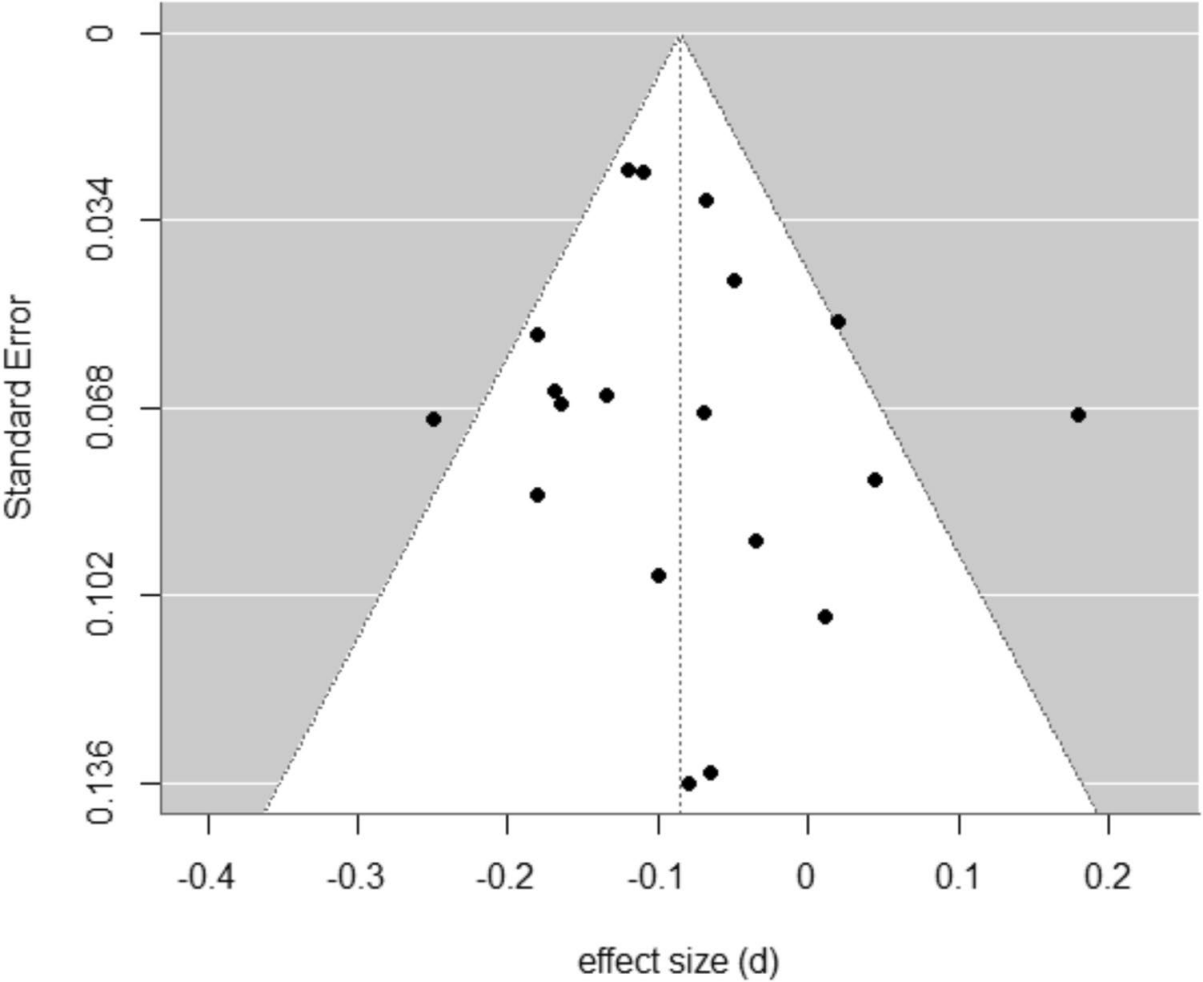
Funnel plot for studies examining age and avoidant decision-making

Visual inspection of a funnel plot with the aggregated within-study effect size estimates and standard errors suggested possible asymmetry, with slightly more studies with effect sizes smaller than the estimate particularly with larger standard errors (Fig. 3). However, an Egger’s regression test did not detect any significant bias (*b* = − 0.11, 95% CI [− 0.21, − 0.01], *p* = 0.600). Power analysis was performed using the metapower package (Griffin 2021). Results showed that according to an 80% power convention, we had adequate power to detect a summary effect size (100%). Additionally, we had 99.99% power for a random-effects analysis, with *k* = 41 and an average sample size of 357, with moderate to large heterogeneity (*I*^2^ = 60%).

## Discussion

The current meta-analysis of 19 studies (*N* = 7969) investigated the relationship between age and avoidant decision style, as measured by the avoidant subscale of the GDMS and the buck-passing subscale of the MDMQ (i.e., complete avoidance), as well as the dependent subscale of the GDMS (i.e., partial avoidance). The meta-analytic average correlation between increasing age and less avoidant decision style was small, *r* = − 0.09, yet the lower bound equivalence test was nonsignificant (i.e., the confidence interval [− 0.13, − 0.05] extended beyond *r* = − 0.10), providing support for the practical or theoretical significance of the effect (Lakens 2017). We also found a moderating effect of participant sample type (i.e., university students, professionals, and community samples), the effect size metric (i.e., point-biserial correlation or Pearson’s *r* correlation coefficient), and degree of avoidance (i.e., complete (the avoidant and buck-passing subscales) vs. partial (the dependent subscale)). The meta-analytic average correlation between age and avoidant decision-making style was not moderated by age range, gender, culture, the avoidant decision style subscale, nor publication year.

Our results indicate that older age is associated with a small decrease in avoidant decision-making style, and this association appears specific to complete decision avoidance rather than partial avoidance. While previous research has demonstrated an association between increasing age and more dependent and avoidant decision style, mediated by declines in executive functioning (Fatima et al. 2020), the present results contradict this idea. This may be because cognitive declines with age depend highly on individual factors, such as prior intelligence level (Deary et al., 2009). Although studies included in the present meta-analysis did include measures of cognitive function (i.e., Basu and Dixit, 2022; Calleja et al. 2020; Erceg and Galić, 2024; Fatima et al. 2020; Kornilova et al. 2018; Leon et al., 2025b), the operationalization of cognition differed across these studies (e.g., general cognitive ability, executive functioning, meta-cognition, and inductive reasoning). Given this variability, cognition was not examined as a moderator. This would be an important avenue for future research. Nevertheless, the current findings do not support the idea that typical age-related declines in cognitive functioning are likely to lead to more dependent or avoidant decision styles. Rather, older adults may be more decisive and less avoidant in their decision-making by relying on their lived experience and accumulation of wisdom (Worthy et al. 2011).

Another factor that may influence avoidant decision-making in older age is whether the decision-maker is focused on outcomes, including anticipated negative outcomes (Frank and Kong 2008). Older adults may be more likely to engage in decision avoidance as an emotion regulation strategy when forced into situations that can cause unwanted negative emotions (English and Growney 2021). Indeed, emotion regulation strategies have been associated with different decision-making styles. For example, a suppression-based regulation strategy is associated with an avoidant decision style (Farokhi and Hosseinchari 2020). Emotion regulation strategies affect decision-making indirectly by influencing the timing, experience, and expression of one’s emotions (Grecucci and Sanfey 2014). As such, it has been argued that due to age-related changes in emotional processes and goals, it is vital for research investigating age and decision-making to also consider emotion (Mikels and Taullahu 2023). Specifically, ongoing emotion can affect decision-making (Loewenstein and Lerner 2009) by contributing toward determination of goals, and directing attention to meet these goals (Hanoch et al. 2007). Emotions also drive behavior toward rewards and away from punishments (Mitchell 2011). In line with this, older adults who make risky decisions do so in anticipation of positive emotions associated with the decision outcome (Chen and Ma 2009). Future research is needed to investigate the extent that cognitive versus emotional processes may underpin changes in decision style in older age. It would also be important to examine whether lesser avoidant decision style among older adults is associated with more positive expectations regarding decision outcomes.

Older adults have reported preferring to make important decisions alone, which is related to a less avoidant decision profile (Delaney et al. 2015). Since the GDMS questionnaire specifically asks about ‘important’ decisions, it might be suggested that the current findings are specific to important decisions only. However, the word ‘important’ was removed from the GDMS in two of the studies included in the current meta-analysis (i.e., Leon et al. 2025a, b), and is not included in the MDMQ. Furthermore, no difference was identified between the GDMS and MDMQ subscales in moderator analysis. It is therefore unlikely that decision importance explains the overall lesser avoidant decision style among older adults in the current data. Nevertheless, future research should directly test whether decision importance, or other aspects of the decision context, influence the correlation between age and avoidant decision style.

Avoidant decision-making style is associated with lesser decision-making competence, while the rational decision-making style is related to greater decision-making competence (Bruine de Bruin et al. 2007). Moreover, scores on the avoidant decision-making style subscale of the GDMS are negatively associated with scores on the rational decision-making style subscale (Bavolar and Bacikova‐Sleskova, 2020). Similarly, the vigilance subscale of the MDMQ represents an adaptive decision-making profile, while the buck-passing subscale contributes toward maladaptive decision-making (Mann et al. 1997). It would be worthwhile for a future meta-analysis to extend the current study to include the full range of decision-making styles measured by the GDMS and MDMQ. Such examination may reveal whether there is a relationship between age and the rational/vigilant style and whether this co-occurs with a negative association between age and the avoidant style. This may help to elucidate whether decision styles in older adulthood are adaptive.

The effect size metric was identified as a moderator of the association between older age and less avoidant decision style. This suggests that a negative association between age and avoidant decision-making style may be driven by effect sizes calculated from Pearson’s *r* correlation coefficients, relative to those calculated from point-biserial correlation. However, we treat this difference with caution as the majority of effect sizes included within the present meta-analysis provided Pearson’s correlation coefficients. The moderator analyses also showed that community samples, rather than university and professional samples, demonstrated an association between older age and less avoidant decision-making style. Given the university and professional samples typically involved young to middle-aged adults (i.e., 16–50 years), any decrease in avoidant decision style may not occur until later in life. Together with the moderation by degree of avoidance, this suggests that among older adults, a less avoidant decision-making style may be specific to complete decision avoidance, such as putting off decisions, and leaving them to others.

In line with the call to consider group and cultural differences in decision-making research (Appelt et al. 2011), the current meta-analysis extracted Hofstede’s (2001) national culture dimensions relevant to each study’s sample. As there was no evidence that these dimensions moderate the relationship between age and avoidant decision-making style, the results may be robust across a variety of social frameworks and cultures. Because culture was scored based on the country the study took place in, it is possible that some participants had different cultural backgrounds than the predominant culture in that country. Alternatively, there may be other subcultural/group-related traits or shared experiences that moderate an association between age and avoidant decision style. For example, Calleja et al. (2020) reported that among military officers who had better tactical planning performance, there was greater intuitive decision-making style. This may relate to Hofstede’s (1998) proposition that subcultures exist within the context of organizations. Further investigation is needed to understand whether subcultures or groups influence age and avoidant decision-making. Given that subcultures can exist in job types or job classes (Marzec 2018), capturing the current (or previous for older adults) employment status/careers of participants, representative of this type of subculture, may reveal a moderator of age and decision style associations.

It is important to note limitations of the current meta-analysis. Firstly, we chose to use self-reported decision-making styles as a measure of avoidant decision-making. It is possible that an investigation of studies using behavioral/actual decision-making tasks (see, for example, Deng et al. 2022) may reveal different results. Thus, the current research should be extended to examine the association between age and behavioral decision-making tasks that measure partial or complete decision avoidance. Additionally, decision-making questionnaires rely on explicit and conscious knowledge of decision-making and may therefore be vulnerable to biases such as social desirability (Balconi et al. 2023). The cross-sectional samples in the current research also preclude any conclusions relating to effects of aging as opposed to age-related differences in decision style, with the latter potentially confounded by cohort effects (Lindenberger et al. 2011). Although given that publication year of the studies was not a moderator of the association between age and avoidant decision-making, it is possible that the current finding might be attributable to effects of aging rather than cohort effects.

Consistent with Bagaini et al. (2023), there was no moderation by age range, suggesting that a wider age range does not increase power to detect age differences. However, it is possible that participants in the studies were not evenly distributed across the adult age range. A recommendation for future research is to provide data specific to each decade age bracket to ensure that potential effects of middle-age decision style can be distinguished from the effects of young and older adulthood. A further recommendation is to broaden the conceptualization of older age to examine how factors such as life transitions (e.g., retirement, bereavement, grandparenthood; Bytheway, 2005), subjective age (Alonso Debrecenzi and Bailey, 2021), or residence (e.g., independent living versus care setting; Bytheway, 2005) influence decision style. Future research should also examine the potential influence of older age on indecisiveness, as measured by Frost and Shows’ (1993) Indecisiveness Scale. To the best of our knowledge, the indecisiveness literature to date has focused on young adults (e.g., Lauderdale et al. 2024; Rassin et al. 2007). Since aversive (and not avoidant) indecisiveness is associated with negative affectivity during decision-making (Spunt et al. 2009), future studies would potentially identify differential effects of older age on aversive indecisiveness relative to avoidant indecisiveness.

## Conclusions

Evidence for the proposition that older age is associated with avoidant decision-making is mixed (e.g., Delaney et al. 2015; Fatima et al. 2020). The present systematic review and meta-analysis provides support against this proposition, indicating a decrease in avoidant decision-making style with age. Furthermore, this decrease appears specific to complete, and not partial, decision avoidance. While small, the effect appears robust across publication year (and potentially cohort effects), age range, type of avoidant decision style measure, gender, and culture. The results also indicate that the effect is nonsignificant among young to middle-aged student and professional samples, suggesting that decision style may not become less avoidant until later in life. Further research is needed to understand the significant heterogeneity in the association between age and avoidant decision style, with variables such as decision importance, self-perceptions of decision-making ability, emotions associated with expected decision outcomes, and the inclusion of different subcultures, likely to provide insight. This initial synthesis of findings provides a useful roadmap for future research.

## Supplementary information

Below is the link to the electronic supplementary material.

Supplementary file1 (DOCX 14 kb)

## Data Availability

All materials, data, and analysis code related to this manuscript will be made publicly available. The study was preregistered through AsPredicted prior to data extraction and analysis https://aspredicted.org/3nd5-t95x.pdf, and data are available at https://osf.io/6vrmh/.
