# Supplementary material for: A systematic review and meta-analysis of the association between age and degrees of avoidant decision-making style
Source: Eur J Ageing. 2025 Oct 15;22(1):50. doi: 10.1007/s10433-025-00887-5 (PMC12528634; doi:10.1007/s10433-025-00887-5)
Supplement: Supplementary file 1 — Supplementary file1 (DOCX 14 kb) [file 10433_2025_887_MOESM1_ESM.docx]

Supplementary Materials

Results

**Overall Pooled Effect**

***Non-Significant Moderators***

Age difference (ranging from 3 to 75 years) was not a significant moderator of the association between age and avoidant decision-making (*b* = < 0.001, 95% CI [-0.003, 0.003], *p* = .964). The proportion of females in each study (ranging from 0% to 100%) was also not a significant moderator (*b* = -0.001, 95% CI [-0.003, 0.001], *p* = .298). For each dimension of culture: power distance (ranging from 38 to 93) was not a significant moderator (*b* = -0.001, 95% CI [-0.005, 0.003], *p* = .666); individualism (ranging from 5 to 100) was not a significant moderator (*b* = -0.002, 95% CI [-0.008, 0.005], *p* = .427); motivation towards achievement and success (ranging from 14 to 62) was not a significant moderator (*b* = 0.001, 95% CI [-0.003, 0.005], *p* = .571); uncertainty avoidance (ranging from 46 to 95) was not a significant moderator (*b* = 0.001, 95% CI [-0.001, 0.003], *p* = .488); long-term orientation (ranging from 19 to 67) was not a significant moderator (*b* = -0.001, 95% CI [-0.01, 0.01], *p* = .852); and, indulgence (ranging from 0 to 71) was not a significant moderator (*b* = -0.001, 95% CI [-0.005, 0.004], *p* = .785). There were no influences of the MDMQ buck-passing (*k* = 5) or GDMS dependent (*k* = 18) decision style subscales, relative to the GDMS avoidant (*k* = 18) decision style subscale (*b* = 0.09, 95% CI [-0.08, 0.25], *p* = .226, and *b* = 0.08, 95% CI [-0.09, 0.24], *p* = .287, respectively). Publication year (ranging from 2000 to 2024) was not a significant moderator (*b* = -0.004, 95% CI [-0.02, 0.01], *p* = .411).
